# Evaluation of fig‐milk dessert bioactive properties as a potential functional food

**DOI:** 10.1002/fsn3.3950

**Published:** 2024-01-09

**Authors:** Niloofar Zare, Mahsa Sedighi, Hasan Jalili, Hamid Zare, Neda Maftoon Azad

**Affiliations:** ^1^ Department of Life Science Engineering, Faculty of New Sciences and Technologies University of Tehran Tehran Iran; ^2^ Department of Pharmaceutics and Nanotechnology, School of Pharmacy Birjand University of Medical Sciences Birjand Iran; ^3^ Cellular and Molecular Research Center Birjand University of Medical Sciences Birjand Iran; ^4^ Fig Research Station, Fars Agricultural and Natural Resources Research and Education Center, AREEO Estahban Iran; ^5^ Agricultural Engineering Research Department, Fars Agricultural and Natural Resources Research and Education Center Agricultural Research, Education and Extension Organization (AREEO) Shiraz Iran

**Keywords:** anticancer activity, antioxidant activity, bioactive substances, fig‐milk dessert, sensory properties

## Abstract

The fig‐milk dessert, a traditional and nutritionally rich treat infused with bioactive compounds, was subjected to a comprehensive analysis in this study. The novelty of this research lies in the investigation of the in vitro antioxidant, anticancer, and antimicrobial potential of the fig‐milk dessert. This was accomplished through the utilization of the 2,2‐diphenyl‐1‐picrylhydrazyl (DPPH) assay, Annexin/propidium iodide staining, microtiter plate‐based assay and agar well diffusion, respectively, for the first time. Additionally, the study assessed the total phenols and flavonoid content of the extract using the Folin–Ciocalteu assay and the aluminum chloride method, respectively. The findings revealed that the cooking method exerted a significant influence on the bioactive properties and nutritional composition of the dessert. Among the samples analyzed, CM1, consisting of figs steamed for 2 min and milk heated to 70°C, exhibited remarkable characteristics. This sample demonstrated the highest peptide concentration (1290 mg/L), superior antioxidant and anticancer activities, and favorable sensory attributes. Specifically, CM1 induced apoptosis in 84% of AGS cells and inhibited 68% of free radicals in the DPPH assay. It is noteworthy that the fig‐milk dessert did not exhibit any antibacterial properties. These discerning results carry substantial implications for the development of functional dairy products endowed with both nutritional and potential therapeutic properties.

## Graphical abstract

Fig‐milk dessert is a traditional nutritious dairy dessert rich in bioactive substances. Findings results could have important implications for the development of functional dairy products with nutritional and potentially therapeutic properties.

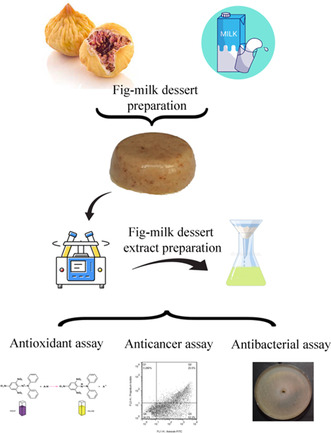

## 1 INTRODUCTION

In recent years, with the persistent escalation of healthcare costs, there has been an increasing demand for health‐conscious diets and lifestyles. In response, the research focus within the food industry has shifted toward functional foods, presenting a promising avenue. Customers are becoming progressively cognizant of the direct impact that food can exert on their health (Bigliardi & Galati, 2013). Functional foods are delineated as either natural or processed food items incorporating bioactive compounds capable of promoting optimal health and mitigating the occurrence of non‐communicable diseases, as posited by the Functional Food Center, these compounds are employed for the prevention, management, and even treatment of chronic diseases (Brown et al., 2018; Martirosyan & Singh, 2015). Functional dairy products stand out as one of the most promising categories within the realm of functional foods, offering potential benefits that span enhanced immune system functionality, antimicrobial activity, diminished cancer risk, improved bone health, and antioxidant defenses against free radical damage. Over recent years, a diverse array of beneficial dairy products has been globally introduced, containing bioactive peptides, dietary fiber, flavonoids, carotenoids, conjugated linoleic acids, antioxidants, oligosaccharides, calcium, probiotics, and other compounds renowned for their exceptional nutritional and therapeutic properties (Yildiz & Ozcan, 2019). The infusion of fruits and vegetables into dairy products presents an attractive avenue for fortification, given the vibrant colors, flavors, aromas, fiber, and vitamin content inherent in these natural sources. This integration serves to augment the nutritional profile and bioactivity of dairy products (Farooq et al., 2023; Mousavi et al., 2019; Salehi, 2021). Fig‐based products have an extensive history of utilization in traditional medicine to treat a variety of ailments. Consequently, there has been a burgeoning consumer demand for both fig fruit and fig‐based products in recent decades (Rasool et al., 2023; Suttisansanee et al., 2021; Teruel‐Andreu et al., 2021). As an example, the extract from *Ficus awkeotsang* Makino exhibited antioxidant and anti‐inflammatory activities on Hs68 fibroblasts and BALB/c mice. Finding from studies on this extract suggested its potential as an adjuvant in pharmaceutical applications, particularly against damages induced by free radical (Lin et al., 2022). Fig powder was employed as a natural sweetener and flavoring supplement in the formulation of goat's milk yogurt, resulting in elevated total acids, carbohydrate contents, and total lactic acid bacteria count. Observations indicated that fig powder holds significant potential for enhancing the taste, aroma, and texture of yogurt, effectively masking any undesirable flavors inherent in milk (Mahmoudi et al., 2021).

Milk‐based desserts exhibit a broad spectrum of textures, tastes, and appearances, appealing to consumers globally. Nevertheless, a substantial number of these desserts are known for their elevated levels of fat and sugar, potentially leading to adverse health effects. In recent years, diabetes has emerged as a leading cause of death globally, with the World Health Organization reporting 1.5 million deaths attributed to diabetes in 2019. Considering the health risks associated with high‐fat and high‐sugar diets, there is a crucial imperative to create desserts and other food products that not only deliver an enjoyable taste but also contribute to consumer well‐being (Jahromi & Niakousari, 2018). In a study conducted by Jahormi and colleagues, an industrial recipe for producing fig‐milk dessert was introduced, incorporating carboxymethyl cellulose (CMC) into the conventional formulation. The investigation revealed that steaming the figs for 10 min and subsequently blending them with high‐temperature milk (90°C) successfully mitigated the bitterness associated with the dessert (Jahromi & Niakousari, 2018).

Phenolic compounds, among the diverse phytochemical constituents present in figs, stand out as the primary antioxidants. These compounds exhibit various functionalities, serving as reducing agents, hydrogen donors, free radical scavengers, singlet oxygen scavengers, and more. Studies have indicated that the consumption of figs can substantially enhance the antioxidant capacity of plasma, persisting for up to 4 h after ingestion. This effect is particularly noteworthy in counteracting the oxidative stress induced by the consumption of high‐fructose corn syrup in carbonated soft drinks (Caliskan, 2015). As per the findings of Vinson et al., the consumption of 40 g of dried figs has been demonstrated to augment plasma antioxidant capacity (Vinson et al., 2013). Furthermore, Faleh et al. illustrated that figs possess the capability to neutralize free radicals (Faleh et al., 2012). Figs harbor diverse compounds with demonstrated anticancer properties. Notably, quercetin, a constituent of figs, promotes apoptosis in Caco‐2, HT‐29, and HL‐60 leukemia cells by triggering the release of cytochrome c from mitochondria. Moreover, quercetin synergistically operates with the chemotherapeutic agent Cisplatin, impeding protein kinase C. Additionally, flavonoid compounds in figs, including lutein, exhibit the capacity to inhibit angiogenesis and induce apoptosis in cancer cells (Purnamasari et al., 2019). In addition to quercetin and lutein, figs encompass various other compounds with demonstrated anticancer properties, including benzaldehyde and coumarins. Benzaldehyde, notably, has shown effectiveness in the treatment of terminal human carcinoma (Caliskan, 2015). It has been reported that the total extract of the *Ficus carica* L. exhibits dose‐dependent antiproliferative activity and photodynamic cytotoxicity against the C32 cell line (Marrelli et al., 2012).

The biological activities of fig‐milk dessert are predominantly attributed to the phytochemical compounds in figs and the peptides generated through the proteolysis of milk proteins. Recently, there has been a heightened interest in employing suitable cooking methods to preserve the active compounds and nutrients within food products (Palermo et al., 2014; Roy et al., 2007). Hence, the objective of this study was to quantify the concentration of peptides, total phenols, and total flavonoids in samples of fig‐milk dessert and explore their correlation with variations in biological activities, encompassing antioxidant, anticancer, and antibacterial properties. Additionally, the impact of different cooking methods on nutrient concentration and biological activities was scrutinized to identify the optimal fig‐milk dessert with regard to both sensory and bioactive characteristics.

## 2 MATERIALS AND METHODS

### 2.1 Materials

The formulation employed pasteurized milk sourced from a local market in Shiraz, Iran (Pegah Fars Dairy Co.). The dried figs used in this study were obtained from a sorting and packaging company located in Estahban, Iran (Estahban Anjiran Zarrin Co., Fars, Iran). Chemicals including O‐Phthalaldehyde (OPA), sodium tetrahydridoborate, sodium dodecyl sulfate, and quercetin were purchased from Merk Group Chemical Company. Additionally, 2,2‐diphenyl‐1‐picrylhydrazyl (DPPH), gallic acid (GA), and sodium carbonate were acquired from Sigma‐Aldrich Chemicals Company. All experimental procedures were carried out using deionized water (dH_2_O).

### 2.2 Dessert preparation

To prepare the fig‐milk dessert, a modified protocol based on Jahromi and Niakousari's method was utilized (Jahromi & Niakousari, 2018). In order to eliminate bitterness from the dessert, the figs underwent a steaming process lasting 10 min, after which they were combined with hot milk at a temperature of 90°C. However, considering the sensitivity of fig proteases and active compounds to high temperatures (Caliskan, 2015; Fadýloğlu, 2001), it is crucial to optimize the duration of fig steaming and the temperature of the milk before proceeding to measure nutrient concentrations and bioactivities. For optimization purposes, we varied the milk temperature (4, 25, 40, 50, 70, 80, and 90°C) and the duration of fig steaming (2, 5, 7, 9, and 10 min). A total of 35 samples (Table S1) were prepared and each consisting of 16.5 gr of figs steamed for the specified durations. Subsequently, each steamed fig sample was mixed with 84 mL of milk at the designated temperatures, ensuring homogeneity through 30 seconds of stirring. All 35 fig‐milk samples were stored at 4°C for a duration of 4 h. Following storage, the bitterness and sweetness of each sample were experimentally assessed. Four sweet samples, characterized by the shortest fig steaming times (2 and 5 min) and high milk temperatures (70 and 90°C) were selected and labeled for further analysis (named as CM1‐CM4, Table S2).

### 2.3 Preparation of aquatic extract of fig‐milk dessert

For each fig‐milk sample, 20–25 g were carefully dispensed into a 50‐mL falcon tube and subjected to centrifugation at 4000 × *g* for 30 min at 4°C. The ensuing supernatant was meticulously filtered using Whatman filter paper and subsequently transferred into 2‐mL vials. The solution underwent an additional round of centrifugation at 14,000 × *g* for 30 min. To ensure the exclusion of microbial contamination and achieve a clear aqueous extract, the resulting supernatant was carefully passed through a 0.22‐μm membrane filter, following established protocols (Elfahri et al., 2016; Lee et al., 2020; Shori, 2020). The resultant filtrate was then utilized for subsequent experiments.

### 2.4 Determination of peptide concentration

To ascertain peptide concentration, the OPA method was employed, involving the measurement of the free amino groups. To execute this, a solution was prepared by combining 25 mL of 100 mM sodium tetrahydroborate solution, 2.5 mL of 20% v/v sodium dodecyl sulfate (SDS), 1 mL of OPA solution (40 mg OPA in 1 mL methanol), and 50 mL of dH_2_O. It is important to note that the OPA solution was freshly prepared on a daily basis. Next, 50 μL of the aqueous extract of the fig‐milk dessert was mixed with 1 mL of the OPA solution in a quartz cuvette. Following a 2‐min incubation period in darkness, the absorbance of the mixture was measured at 340 nm. A calibration curve was established using peptone as a reference standard (Darvish et al., 2017; Sadeghi et al., 2018; Sedighi et al., 2016).

### 2.5 Measurement of total phenols

To determine the total phenols concentration of the aqueous extract, the Folin–Ciocalteu reagent (Sigma, ST. Louis, MO) was employed. The procedure involves the use of 7.5% sodium carbonate and 10% Folin–Ciocalteu reagent. In this method, 500 μL of the aqueous extract solution was mixed with 2.5 mL of Folin–Ciocalteu reagent and 2 mL of sodium carbonate. After storage at room temperature (RT) for 1 h, the sample's absorbance was measured at 765 nm. A calibration curve was created using GA as a reference standard (Moein et al., 2015).

### 2.6 Measurement of total flavonoids

The determination of total flavonoid concentration was carried out using a 2% aluminum chloride solution. To perform this analysis, 1.5 mL of the aqueous extract and 1.5 mL of the 2% aluminum chloride solution were combined in a test tube. After allowing the mixture to stand at RT for 1 h, the absorbance of the sample was measured at 415 nm and converted to flavonoid content based on the quercetin calibration curve (Quettier‐Deleu et al., 2000).

### 2.7 Determination of antioxidant activity

The antioxidant activity was assessed using the DPPH assay. Briefly, 800 μL of a 0.1 mM DPPH solution was combined with 200 μL of each aqueous extract, shaken vigorously, and then incubated for 30 min in the dark at RT. Following incubation, the absorbance of samples was measured at 517 nm using a UV–Vis spectrophotometer. The antioxidant activity was calculated using the following equation (Elfahri et al., 2016; Tavallali & Zareiyan, 2018):

$$\text{Radical scavenging activity}\;\left(\%\right)=100\times \left(1-\left(\frac{\text{Sample}-\text{Blank}\;}{\text{Control}\;}\right)\right)$$

Where the blank consisted of a mixture of 20 μL of extract and 200 μL of methanol, and control was composed of 20 μL of methanol and 200 μL of DPPH solution. The positive control used in the experiment was GA, which had a concentration of 25.4 mg/L. All experiments were performed in triplicate.

### 2.8 Cell culture

The AGS cells (a human gastric adenocarcinoma cell line) were obtained from the American Type Culture Collection (ATCC, Manassas, VA, USA). The AGS cells were cultured in RPMI1640 medium (Gibco medium), supplemented with 10% sterile‐filtered fetal bovine serum (FBS) and a 1% antibiotic solution (l‐glutamine‐penicillin–streptomycin) (Sigma‐Aldrich, Germany). The cell culture was maintained in a humidified incubator at 37°C with 5% CO_2_.

### 2.9 Annexin V/propidium iodide assay

The Annexin V‐FITC assay was performed using the Annexin V‐FITC assay kit (Sigma‐Aldrich) according to the manufacturer's recommended protocols. Specifically, 2 × 10^5^ AGS cells were treated with the aqueous extract for 24 h, while negative control cells were cultured without any treatment. Subsequently, the cells were detached using trypsin and centrifuged at 300 × *g* for 10 min. They were then washed three times with phosphate‐buffered saline (PBS) to remove any residual medium. The cells were stained using an apoptosis detection kit, washed once with PBS buffer, and then once with 1000 μL X‐binding buffer. Next, the cells were suspended in 100 μL of binding buffer containing 5 μL Annexin V‐FITC for 15 min. Afterward, the cells were washed again with 1000 μL of binding buffer and re‐suspended in 200 μL binding buffer containing 5 μL propidium iodide (PI). Finally, the percentage of apoptosis induction was measured using flow cytometry (Kntayya et al., 2018). In this experiment, the positive control utilized was fluorouracil (5‐fu) at a concentration of 45 mg/L.

### 2.10 Determination of antibacterial activity

The impact of the fig‐milk aqueous extract on bacterial growth was investigated using two methods: a microtiter plate‐based assay and agar well diffusion on gram‐negative bacteria, *Bacillus cereus* 11,778 and *Escherichia coli* 13,706.

The microtiter‐based antibacterial assay, 1 mL of *B. cereus* and *E. coli* bacterial stocks were individually cultured in Mueller Hinton (MH) broth medium at 37°C in an incubator for 1 day. Following cultivation, the concentration of the bacterial suspension was adjusted to 0.5 McFarland. To assess bacterial growth in the presence of the aqueous extract from fig‐milk desserts, 100 μL of each sample was mixed with 100 μL of each target bacteria suspension in a 96‐microwell plate. The plates were then incubated at 37°C for 24 h. After the incubation period, the absorbance was measured at 600 nm (Aguilar‐Toalá et al., 2017; Darvish et al., 2017).

To conduct the agar well diffusion assay, bacteria were cultured in a MH broth medium at 37°C. Upon reaching maturity, overnight cultures were diluted to approximately 6.0 log CFU/mL and subsequently spread onto MH agar plate using 100 μL of the bacterial suspension. Next, wells with an 8 mm diameter were created on the agar plates using a sterile stainless‐steel borer. A 100 μL of the aqueous extract was introduced into the wells of the inoculated MH agar plates. The plates were then incubated at 37°C for 24 h, and the antibacterial activity was evaluated by measuring the diameter of the growth inhibition zones (Abdel‐Hamid et al., 2020).

### 2.11 Sensory evaluation of fig‐milk dessert

The analysis of the fig‐milk dessert samples was conducted following the modified method outlined by Granato et al. (2010). Four samples were evaluated by a panel of 14 semi‐trained panelists (9 men and 5 women) from the Agricultural Research and Training Center of Fars Province. The panelists used a 5‐point hedonic scale, ranging from very bad (1) to very good (5), to rate the samples. To initiate the evaluation, 20 g of each sample were placed in individual plastic cups and randomly coded with numbers. These coded samples, along with evaluation forms, were distributed to the panelists. The assessment encompassed appearance and color, taste (including both taste and smell), texture, and general acceptance (Jahromi & Niakousari, 2018). Each sample was tested in triplicate and presented to the same panelist.

### 2.12 Statistical analysis

The measurements were conducted in triplicate, and the results were reported as the mean ± standard deviation of three repetitions. The influence of milk temperature and the duration of fig steaming on the concentration of nutrients and biological activities in the fig‐milk dessert were analyzed through a two‐way analysis of variance (ANOVA) with a significance level of 5%. Tukey's test was then employed to compare mean differences at the same significance level. All statistical analyses were performed using SPSS 26 software.

## 3 RESULTS AND DISCUSSION

The optimization of fig steaming duration and the milk temperature aimed to identify the most effective preparation method for the fig‐milk dessert. Preliminary findings suggested that steaming figs for a minimum of 2 min and using milk at temperatures exceeding 70°C successfully prevented the development of a bitter taste in the dessert (see Table S1). However, considering the potential detrimental effects of high temperatures on the proteases and active components in fig‐milk dessert (Caliskan, 2015; Fadýloğlu, 2001), it became imperative to minimize the fig steaming duration and milk temperature to control the bitterness. The results presented in Table S1 demonstrated that the shortest fig steaming duration (2 min) at a milk temperature of 70°C could be applied to prepare sweet desserts. Nevertheless, for comprehensive testing of biological properties and assessment of the dessert's composition, it was necessary to select samples prepared with longer durations of fig steaming (5 min) and higher milk temperature (90°C). Consequently, four sweet dessert samples, prepared using the minimum fig steaming duration (2 and 5 min) and higher milk temperatures (70 and 90°C), were selected for further experimentation (see Table S2 and Figure S1).

### 3.1 Peptide concentration

**FIGURE 1:**
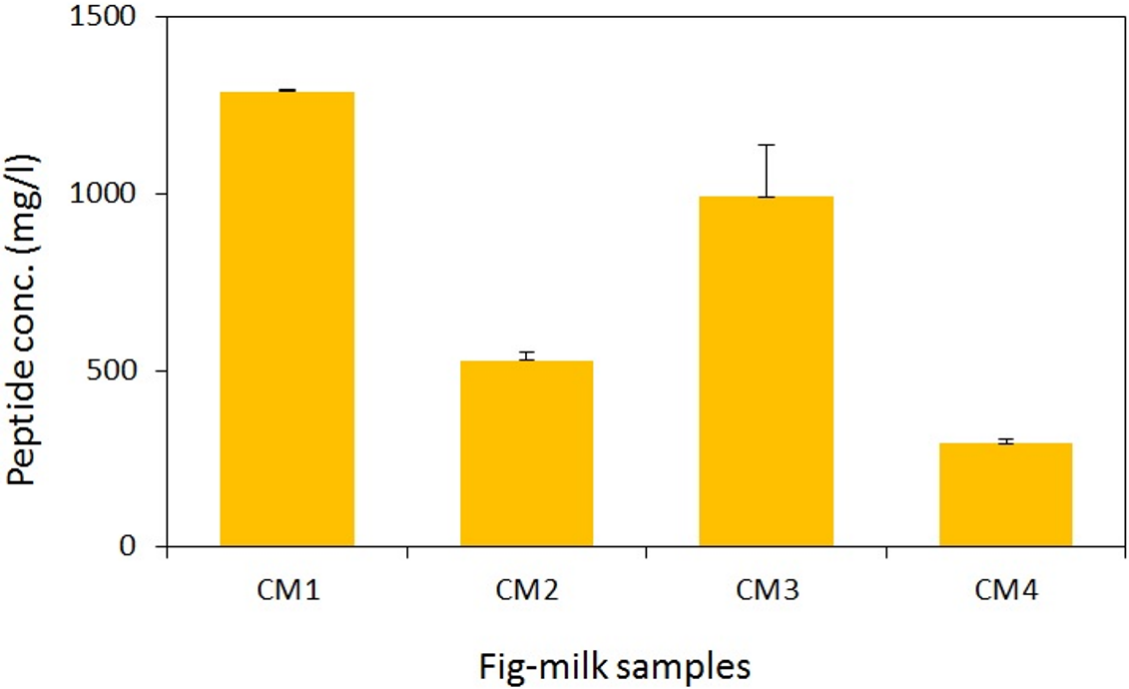
Peptide concentration of aqueous extract of fig‐milk desserts. Values are means ± SD, *n* = 3.

The OPA method was employed to quantify the peptide concentration in the samples. The findings revealed a significant reduction in peptide concentration with an increase in both milk temperature and the duration of fig steaming (*p* < .05). Notably, the interaction between these two variables (milk temperature and fig steaming duration) did not yield a significant impact on the peptide concentration of the desserts. Among the samples, CM1 exhibited the highest peptide concentration (1290 mg/L), whereas sample CM4 displayed the lowest concentration (292 mg/L) (*p* < .05) (Figure 1), underscoring the influence of milk temperature on the peptide concentration.

The observed decline in peptide concentration in the samples is likely attributed to the reduction in fig protease concentration. Existing research indicated that approximately 90% of fig proteases are ficin enzymes, with their optimal activity temperature being 60°C (Fadýloğlu, 2001). The process of steaming figs at high temperatures and combining them with hot milk may lead to the denaturation of the three‐dimensional structure of ficin, resulting in a decrease in ficin concentration. Consequently, as the duration of fig steaming increases from 2 to 5 min and the milk temperature rises from 70 to 90°C, the peptide concentration of the samples decreases. This phenomenon provides a plausible explanation for the disappearance of the bitter taste in the prepared dessert when figs are steamed for at least 2 min and the milk is heated to at least 70°C. Studies have shown that proteolytic hydrolysis of proteins can lead to the formation of peptides with a bitter taste. Peptides with a bitter taste typically contain phenylalanine and tyrosine in their sequence or have hydrophobic amino acids at the C‐terminus or basic and bulky residues at the N‐terminus (Iwaniak et al., 2016; Kilara & Panyam, 2003; Kim & Li‐Chan, 2006; Pripp & Ardö, 2007). Ficin, in particular, possesses the ability to cleave polypeptides at various amino acid sites, especially those in proximity to aromatic and hydrophobic amino acids, resulting in the generation of bitter peptides (Iwaniak et al., 2020). Thus, the reduction in ficin concentration at high temperatures can contribute to a lower concentration of bitter peptides.

### 3.2 Total phenols and flavonoids concentration

**FIGURE 2:**
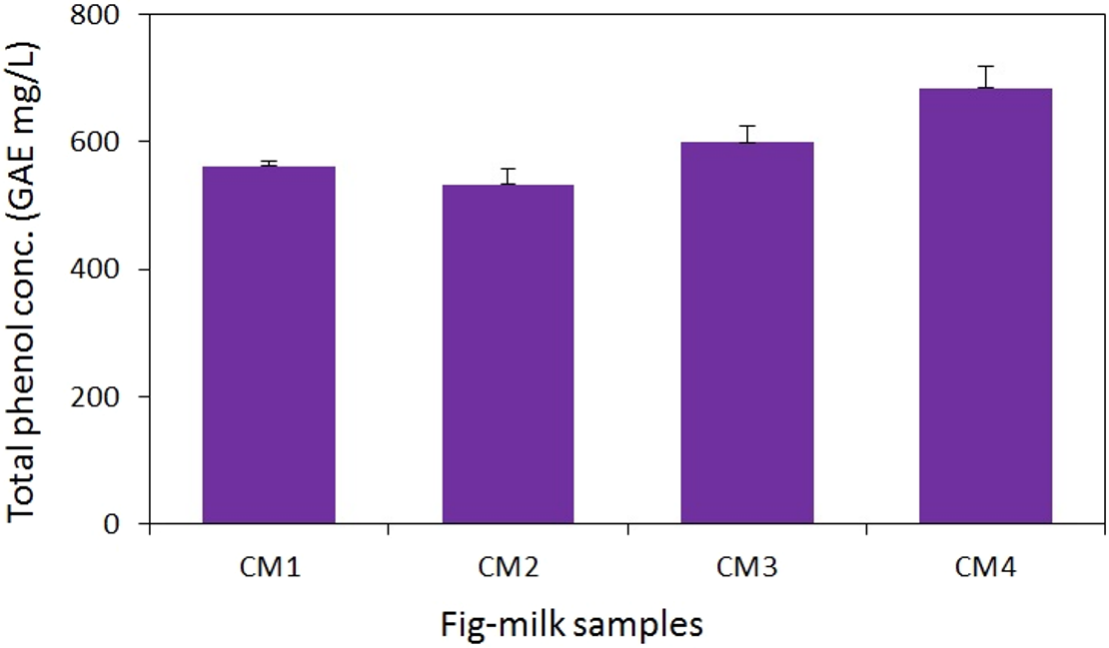
Total phenol concentration in the aqueous extract of fig‐milk desserts. Values are means ± SD, *n* = 3.

The utilization of Folin–Ciocalteu reagent to measure total phenols concentration revealed that elevating the temperature of milk has the potential to increase the total phenols concentration in all selected samples (*p* < .05), while extending the duration of fig steaming did not yield any significant impact. The interaction between the duration of fig steaming time and milk temperature significantly influenced the total phenols concentration of the desserts (*p* < .05), as illustrated in Figure 2. Therefore, samples prepared with 90°C milk (CM3 and CM4) exhibited a significant difference, whereas no significant distinction was observed between samples prepared with 70°C milk (CM1 and CM2). According to the GA calibration curve, CM4 and CM2 samples displayed the highest (684 mgGAE/L) and lowest (533 mgGAE/L) total phenols concentrations, respectively (*p* < .05) (Figure 2).

**FIGURE 3:**
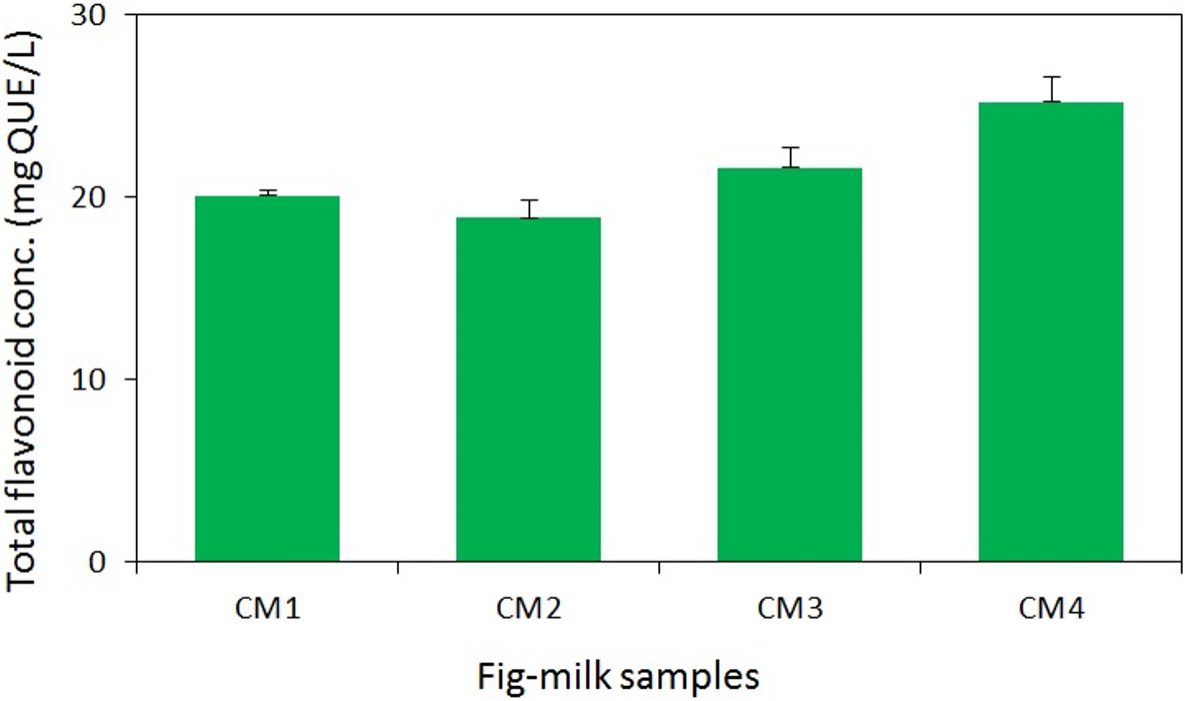
Total flavonoid concentration in aqueous extract of fig‐milk desserts. Values are means ± SD, *n* = 3.

Furthermore, the application of a 2% aluminum chloride solution for measuring total flavonoid concentration revealed that an increase in milk temperature resulted in an elevated total flavonoid concentration in the samples (*p* < .05), while extending the duration of fig steaming did not yield a significant impact. The interaction between the duration of fig steaming and milk temperature significantly influenced the flavonoid concentration of all desserts (*p* < .05), as depicted in Figure 3. Consequently, samples prepared with 90°C milk (CM3 and CM4) exhibited a significant difference, whereas no significant difference was observed between samples prepared with 70°C milk (CM1 and CM2). According to the results, CM4 and CM2 samples displayed the highest (25 mgQUE/L) and lowest (18 mgQUE/L) total flavonoid concentrations, respectively (*p* < .05) (Figure 3).

Phenols and flavonoids are distributed across various organelles and compartments within plants, including cell walls, plasma membranes, and cell organelles, or freely dispersed in the cytoplasm. Food processing techniques involving high temperatures or freezing can induce the release of these compounds, enhancing their extraction potential (Minatel et al., 2017). The findings of this study highlight the impact of milk temperature and fig steaming duration on the concentrations of total phenols and flavonoids in the aqueous extract of fig‐milk desserts. This influence can be attributed to the softening effect of fig steaming on the figs' texture, coupled with the ability of high‐temperature milk to facilitate the release of phenols and flavonoid compounds from the figs. In comparison with other fig‐based products, the fig‐milk dessert exhibited a significantly higher total phenols concentration. In the current study, the phenols concentration in the fig‐milk dessert ranged from 530 mgGAE/L (294 mgGAE/kg) to 680 mgGAE/L (377 mgGAE/kg). This range exceeded the total phenols concentration of fresh fig jam (130–291 mgGAE/kg) and closely approached the total phenols concentration of fermented fresh fig products (530–760 mgGAE/L) (Teruel‐Andreu et al., 2021). Additionally, the total phenols concentration of the fig‐milk dessert stood out when compared to other fortified milk products. Previous studies measuring the total phenols concentration of yogurt, cheese, and ice cream fortified with fruits and vegetables have reported a wide range (50–1500 mgGAE/kg), varying based on the type and concentration of the fortifying substances (Salehi, 2021; Soukoulis et al., 2014).

### 3.3 Antioxidant activity

**FIGURE 4:**
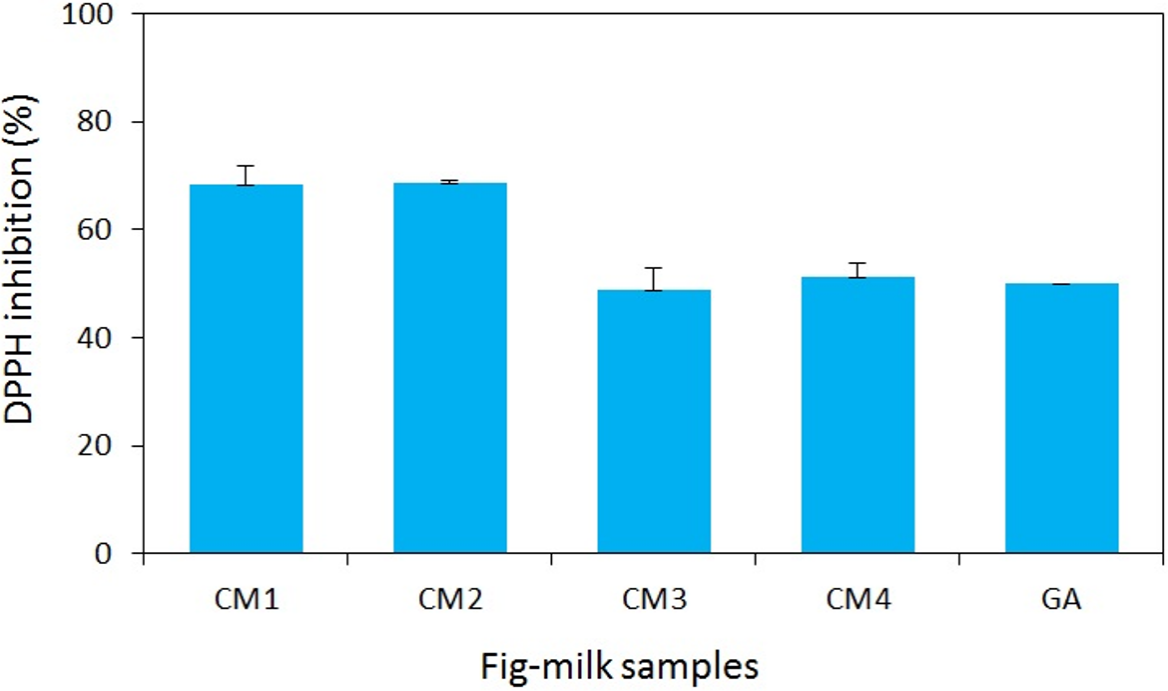
Inhibition of DPPH free radical by the aqueous extract of fig‐milk desserts. Values are means ± SD, *n* = 3.

To assess the antioxidant activity of the fig‐milk desserts, the inhibition of DPPH free radicals was quantified. The results indicated that an increase in milk temperature led to a reduction in the level of free radical inhibition (*p* < .05), while extending the duration of fig steaming did not significantly impact the outcomes. The interaction between the duration of fig steaming and milk temperature did not exert a significant effect on the degree of free radical inhibition. Consequently, samples prepared at the same milk temperature did not exhibit any significant differences (CM1/CM2 and CM3/CM4). The highest percentage of DPPH free radical inhibition was 68%, corresponding to the CM1 sample, whereas the lowest was 48%, associated with the CM3 sample (*p* < .05) (Figure 4).

The investigation revealed that an increase in milk temperature led to a 20% decline in DPPH inhibition. This decrease could be attributed to the destruction of temperature‐sensitive antioxidant compounds present in both milk and figs. The outcomes indicated no observable correlation between alterations in antioxidant activity and changes in peptide, total phenols, or total flavonoid concentrations of the samples.

Certain potent antioxidant compounds in figs, such as quercetin and rutin, are known to be sensitive to high temperatures decomposing when exposed to temperatures as high as 90°C (Buchner et al., 2006). As a result, the CM3 and CM4 samples, prepared with 90°C milk, exhibited lower concentrations of these temperature‐sensitive antioxidant compounds. However, the decrease in the concentration of these compounds did not lead to a reduction in the total phenols and flavonoid contents, as they constitute only a small fraction of the overall phenols and flavonoid compounds in figs (Palermo et al., 2014). The decline in antioxidant activity observed in the CM3 and CM4 samples could also be attributed to the loss of whey protein structure at 90°C. This protein comprises sulfur‐containing amino acids, which can inhibit free radicals (Khan et al., 2019). In a recent study examining the impact of temperature on free radical inhibition during the pasteurization of bovine milk, it was demonstrated that the primary heat treatment of milk (15 s at 65°C) and higher pasteurization temperatures (72–77°C) led to a reduction in the concentration of whey protein in milk, resulting in decreased antioxidant activity (Tripaldi et al., 2021). Several investigations have explored the antioxidant activity of dairy products, including yogurt, cheese, and fortified ice cream, exhibiting a wide range of antioxidant activity (20%–80% inhibition of free radicals) depending on the type and concentration of the natural additives used for fortification (Alenisan et al., 2017; Salehi, 2021; Soukoulis et al., 2014; Yildiz & Ozcan, 2019). Additionally, fresh fig jam and fermented products from fresh figs have been reported to exhibit maximum antioxidant activity of 15% and 30%, respectively (Lu et al., 2021; Rababah et al., 2011).

### 3.4 Anticancer activity

**FIGURE 5:**
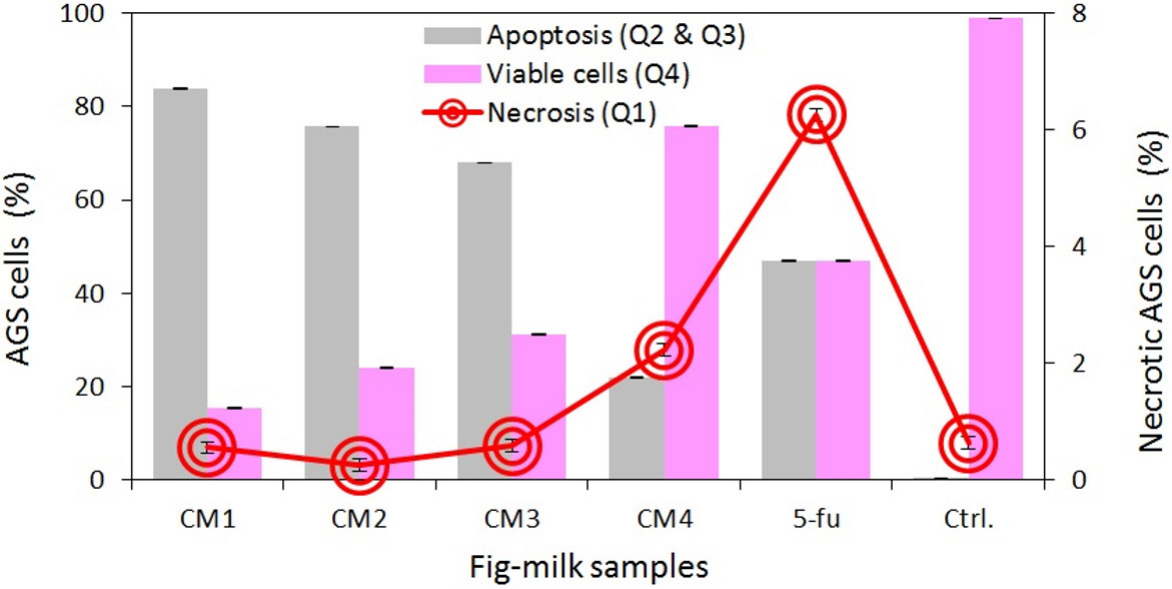
Percentage of apoptosis and necrosis of AGS cells treated by the aqueous extract of fig‐milk desserts. Values are means ± SD, *n* = 3. Ctrl: untreated cells. 5‐fu: fluorouracil.

The annexin V/PI plots delineate the percentage of necrosis and apoptosis induced in AGS cells by various samples of the fig‐milk dessert (see Figure S2). The findings reveal that the aqueous extract of the fig‐milk dessert significantly induced apoptosis in AGS cells, with a low percentage of necrosis. Based on the flow cytometry results, an increase in both milk temperature and the duration of fig steaming led to a decrease in the percentage of AGS cell apoptosis (*p* < .05) (Figure 5). Moreover, the interaction between the two variables had a significant effect on the percentage of AGS cell apoptosis (*p* < .05). The CM1 sample induced the highest apoptosis rate of 84%, while the lowest apoptosis rate of 22% was observed in AGS cells treated with the CM4 sample (*p* < .05) (Figure 5).

Regarding the anticancer activity, the fig‐milk dessert samples (CM1, CM2, and CM3) induced apoptosis in over 65% of AGS cancer cells. However, this effect was observed only at a concentration of 5% aqueous extract of the fig‐milk dessert. Thus, the active substances present in the samples were found to trigger apoptosis in cancer cells at very low concentrations. Given that the bioavailability of peptides and bioactive compounds decreases during the digestive process, the efficacy of the material at low concentrations is crucial (Amigo & Hernández‐Ledesma, 2020). Increasing the duration of fig steaming and milk temperature was found to decrease the rate of apoptosis in AGS cells. The difference in the percentage of apoptosis induced by the CM3 and CM4 samples corresponded to the disparity in their peptide concentration. Furthermore, research has indicated that quercetin and rutin present in figs are the most potent compounds that induce apoptosis, and they are susceptible to decomposition at a temperature of 90°C (Buchner et al., 2006; Purnamasari et al., 2019; Satari et al., 2021; Srivastava et al., 2016). The CM1 and CM2 samples, prepared with milk at 70°C, exhibited higher concentrations of these compounds compared to CM3 and CM4, and therefore had a greater potential to induce apoptosis in AGS cells. Thus, the anticancer activity of fig‐milk desserts primarily relied on the concentration of temperature‐sensitive phytochemical compounds and peptides, while changes in the concentration of total phenol and total flavonoid had no significant effect on the rate of apoptosis induction.

### 3.5 Antibacterial activity

Two methods, the agar well diffusion method and the microtiter‐based antibacterial assay, were employed to assess the antibacterial activity of the aqueous extract. However, the results of both methods demonstrated that the fig‐milk dessert did not hinder the growth of *E. coli* and *B. cereus* bacteria, and hence did not exhibit any antibacterial properties.

Although figs contain compounds that have been reported to impede the growth of bacteria, these compounds possess low solubility in water (Debib et al., 2014). Debib et al. investigated the inhibitory potential of dried fig extracts, including aqueous, ethanolic, acetone, and petroleum ether extracts, on the growth of different bacteria, such as *E. coli* and *B. subtilis*. The study revealed that the aqueous extract of dried figs did not hinder the growth of *E. coli*, but it did impede the growth of *B. subtilis*. In contrast, the extracts prepared with methanol and petroleum ether exhibited inhibitory effects on the growth of *E. coli*. The absence of coumarin and low concentration of tannin in the aqueous extract of dried figs were suggested as possible reasons for this phenomenon. Hence, based on this report, it is plausible to interpret our antibacterial experiment results as the lack of antibacterial compounds in the aqueous extract of fig‐milk desserts.

### 3.6 Sensory properties

The sensory attributes of the fig‐milk desserts, including color and appearance, taste, texture and mouthfeel, and overall acceptability, were evaluated to assess the influence of milk temperature and the duration of fig steaming. The results of the sensory evaluation, presented in Table 1, revealed that all four samples received high scores for all characteristics. The CM1 sample obtained the highest score, while the lowest score was recorded for the CM4 sample. Interestingly, variations in milk temperature and the duration of fig steaming did not lead to any significant changes in the sensory attributes of the product (Table 1).

**TABLE 1:** Sensory evaluation of fig‐milk desserts. All values are mean of triplicate ±SD.

Sample	Color and appearance	Taste	Texture and mouthfeel	General acceptance
CM1	3.78 ± 1.05	3.78 ± 1.05	3.64 ± 0.93	3.64 ± 1.08
CM2	3.57 ± 0.76	3.71 ± 0.73	3.57 ± 0.65	3.64 ± 0.63
CM3	3.92 ± 0.73	3.42 ± 0.76	3.93 ± 0.73	3.64 ± 0.63
CM4	3.35 ± 1.08	3.14 ± 0.95	3.57 ± 0.85	3.28 ± 0.83

In fruit‐based dairy products, color and appearance are crucial parameters that determine the initial quality and acceptability of the product. A visually appealing product can significantly enhance its marketability and consumption (Granato et al., 2010; Jahromi & Niakousari, 2018). Additionally, taste and texture are fundamental attributes that can influence the overall acceptability of the product and serve as primary indicators of initial food acceptance. Our study found that all samples scored high in sensory evaluation, and there were no significant changes in the sensory characteristics of the product due to variations in milk temperature and the duration of fig steaming.

## 4 CONCLUSION

It is concluded that steaming figs for a minimum of 2 min and using milk at a temperature of at least 70°C can effectively control the bitterness of the fig‐milk dessert. Among the four samples evaluated, no significant differences were observed in their sensory properties. Thus, the sample prepared with 70°C milk and figs steamed for 2 min (i.e., CM1), was determined to be the best fig‐milk dessert in terms of taste and bioactive properties. This sample induced apoptosis in 84% of AGS cells and inhibited 68% of DPPH free radicals. Furthermore, the peptide concentration, total phenols concentration, and total flavonoid concentration of the CM1 sample were 1200 mg/L, 562 mg GAE/L, and 20 mg QUE/L, respectively. These findings have the potential to lead to the development of functional dairy products with exceptional nutritional and therapeutic properties. To leverage the bioactive properties of fig‐milk dessert, additional research is necessary to investigate the potential applications of these findings and to develop novel dairy products.

## AUTHOR CONTRIBUTIONS

**Niloofar Zare:** Data curation (equal); formal analysis (equal); investigation (equal); validation (equal). **Mahsa Sedighi:** Data curation (equal); software (equal); writing – original draft (equal); writing – review and editing (equal). **Hasan Jalili:** Conceptualization (lead); data curation (lead); formal analysis (lead); investigation (lead); methodology (lead); project administration (lead); resources (lead); supervision (lead); validation (lead); writing – original draft (lead); writing – review and editing (lead). **Hamid Zare:** Investigation (equal); methodology (equal); supervision (equal); writing – review and editing (equal). **Neda Maftoon Azad:** Investigation (equal); methodology (equal); writing – original draft (equal); writing – review and editing (equal).

## FUNDING INFORMATION

This research did not receive any specific grant from funding agencies in the public, commercial, or not‐for‐profit sectors.

## CONFLICT OF INTEREST STATEMENT

There is no conflict of interest statement in the manuscript.

## Supporting information


Data S1.

## Data Availability

The datasets are available from the corresponding author on reasonable request.
