# Supplementary material for: Evaluation of fig‐milk dessert bioactive properties as a potential functional food
Source: Food Sci Nutr. 2024 Jan 9;12(4):2692–701. doi: 10.1002/fsn3.3950 (PMC11016442; doi:10.1002/fsn3.3950)
Supplement: Supplementary file 1 — Data S1. [file FSN3-12-2692-s001.docx]

**Supplementary Information**

**Evaluation of Fig-milk Dessert Bioactive Properties as a Potential Functional Food**

Niloofar Zare^1^, Mahsa Sedighi^2,3^, Hasan Jalili^1*^, Hamid Zare^4^, and Neda MaftoonAzad^5^

^1^Department of Life Science Engineering, Faculty of New Sciences and Technologies, University of Tehran, Tehran, Iran.

^2^Department of Pharmaceutics and Nanotechnology, School of Pharmacy, Birjand University of Medical Sciences, Birjand, Iran

^3^ Cellular and Molecular Research Center, Birjand University of Medical Sciences, Birjand, Iran

^4^ Fig Research Station, Fars Agricultural and Natural Resources Research and Education Center, AREEO, Estahban 7451877802, Iran

^5^ Agricultural Engineering Research Department, Fars Agricultural and Natural Resources Research and Education Center, Agricultural Research, Education and Extension Organization (AREEO), Shiraz, Iran

^*Corresponding author at: Department of Life Science Engineering, Faculty of New Sciences and Technologies, University of Tehran, Tehran, Iran; (Hasan Jalili) at^ [^hjalili@ut.ac.ir^](mailto:hjalili@ut.ac.ir)

**TABLE S1** Initial assessment of fig-milk desserts based on their taste.

| Sample | Duration of fig steaming (min) | Milk temp. (°C) | Taste | |
| --- | --- | --- | --- | --- |
|  |  |  | Bitter | Sweet |
| 1 | 2 | 4 | * |  |
| 2 | 2 | 25 | * |  |
| 3 | 2 | 40 | * |  |
| 4 | 2 | 50 | * |  |
| 5 | 2 | 70 |  | * |
| 6 | 2 | 80 |  | * |
| 7 | 2 | 90 |  | * |
| 8 | 5 | 4 | * |  |
| 9 | 5 | 25 | * |  |
| 10 | 5 | 40 | * |  |
| 11 | 5 | 50 | * |  |
| 12 | 5 | 70 |  | * |
| 13 | 5 | 80 |  | * |
| 14 | 5 | 90 |  | * |
| 15 | 7 | 4 | * |  |
| 16 | 7 | 25 | * |  |
| 17 | 7 | 40 | * |  |
| 18 | 7 | 50 | * |  |
| 19 | 7 | 70 |  | * |
| 20 | 7 | 80 |  | * |
| 21 | 7 | 90 |  | * |
| 22 | 9 | 4 |  | * |
| 23 | 9 | 25 |  | * |
| 24 | 9 | 40 |  | * |
| 25 | 9 | 50 |  | * |
| 26 | 9 | 70 |  | * |
| 27 | 9 | 80 |  | * |
| 28 | 9 | 90 |  | * |
| 29 | 10 | 4 |  | * |
| 30 | 10 | 25 |  | * |
| 31 | 10 | 40 |  | * |
| 32 | 10 | 50 |  | * |
| 33 | 10 | 70 |  | * |
| 34 | 10 | 80 |  | * |
| 35 | 10 | 90 |  | * |

**TABLE S2** Selected preparation methods for fig-milk desserts.

| Milk temp. (°C) | Duration of fig steaming (min) | Code |
| --- | --- | --- |
| 70 | 2 | CM1 |
| 70 | 5 | CM2 |
| 90 | 2 | CM3 |
| 90 | 5 | CM4 |

*CM: Cooking Method


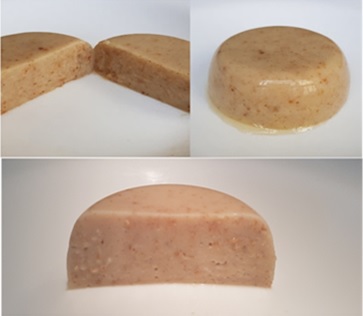


**FIGURE S1** The appearance of selected fig-milk dessert


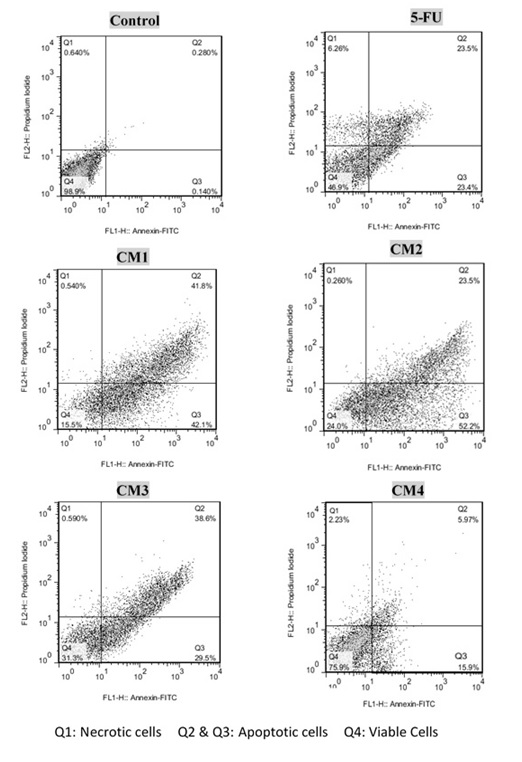


**FIGURE S2** Annexin V/PI assay results of fig-milk dessert by flow cytometry.
